# Anemoside B4 sensitizes human colorectal cancer to fluorouracil-based chemotherapy through src-mediated cell apoptosis

**DOI:** 10.18632/aging.203751

**Published:** 2021-12-10

**Authors:** Xing He, Jun Tang, He-Zhong Yan, Jiao-Xue Wang, Hai-Qing Li, Xiao-Wei Duan, Sen-Yuan Yu, Xi-Lu Hou, Guo-Bin Liao, Wei Liu

**Affiliations:** 1 Department of Gastroenterology, The 901 Hospital of Joint Logistics Support Force, Hefei 230031, Anhui, China

**Keywords:** fluorouracil resistance, colorectal cancer, anemoside B4, Src, caspase-9

## Abstract

Currently, 5-Fluorouracil (5-FU) based chemotherapy is the primary option for colorectal cancer after surgery, whereas chemotherapy resistance related mortality is observed in a large proportion of patients. Anemoside B4 (AB4) is a triterpene saponin, which exhibits a considerable activity in oncotherapy. In this study, we explored the efficacy of AB4 in FU-based chemotherapy in colorectal cancer cells and the underlying molecular mechanisms. Our results indicated a significant synergistic activity of AB4 in 5-FU treated colorectal cancer cells. Furthermore, AB4 treatment eliminated colorectal cancer stem cells by promoting apoptotic cell death in 5-FU resistant colorectal cancer cells. Mechanically, AB4 activated caspase-9 pathway in 5-FU resistant colorectal cancer cells. Elevated Src activity induced cell apoptosis and cancer stem cells elimination effects in AB4 treated colorectal cancer cells. In conclusion, AB4 showed promising sensitization effect in the FU-based chemotherapy of colorectal cancer. Our study may pave a way to ameliorate FU-based chemotherapeutic efficiency in colorectal cancer.

## INTRODUCTION

Despite the advances and optimization in colorectal cancer administration, increasing incidence and mortality has been calculated in the young adults over the last 25 years [1]. Currently, conventional chemotherapy after surgical resection is the first-line chemotherapy regimen for colorectal cancer patients. Among them, 5-Fluorouracil (5-FU) is the primary option for chemotherapy regimens, such as FOLFOX and FOLFORI [2]. However, a large proportion of patients suffer relapse and cancer-related mortality during chemotherapy, which is referred as 5-FU resistance [3]. Further study for the mechanisms of FU resistance is an essential step for improving therapeutic efficiency of colorectal cancer.

Fluorouracil (FU) is an irreversible inhibitor of thymidylate synthase, which interferes with nucleoside metabolism to induce tumor cell death [4]. FU treatment increases the permeability of the outer mitochondrial membrane to induce cell damage [5]. The aggregation and activation of caspase-9 causes DNA damage and cell apoptosis [6]. Caspase-9 has been reported as an initiating caspase for endogenous apoptotic pathway [7]. Dysregulated phosphorylation of the signaling is critically involved in FU-based chemotherapy resistance [8]. Previous reports identified Src kinase phosphorylated caspase-9 to enhance its apoptotic activity [9], which was a promising novel strategy for chemotherapy resistance reversal in colorectal cancer.

Anemoside B4 (AB4) is a main effective triterpene saponin from the roots of *Pulsatilla chinensis* (Bunge) [10]. *P. chinensis* shows a considerable activity on anti-inflammatory and antioxidant [11]. Recent studies also identified the therapeutic potential of triterpenoid saponin AB4 in the treatment of hepatocellular carcinoma and chronic myeloid leukemia [12, 13]. AB4 induces cancer cell apoptosis and autophagy to inhibit cell proliferation and angiogenesis inhibitor [14]. Besides, AB4 attenuates nephrotoxicity of cisplatin during chemotherapy [15], which indicates its potential use in the combined treatment of chemotherapy. Detailed mechanism study was also needed for the effects of AB4 on chemotherapy treatment.

In the present study, we determined the effects of AB4 in FU-resistant colorectal cancer cells. AB4 sensitizes colorectal cancer to 5-FU-based chemotherapy by elevated Src activation and caspase-9 apoptotic pathway. Our study may pave a way to ameliorate FU-based chemotherapeutic efficiency in colorectal cancer.

## RESULTS

### AB4 sensitizes colorectal cancer cells to 5-FU treatment *in vitro*

**Figure 1:**
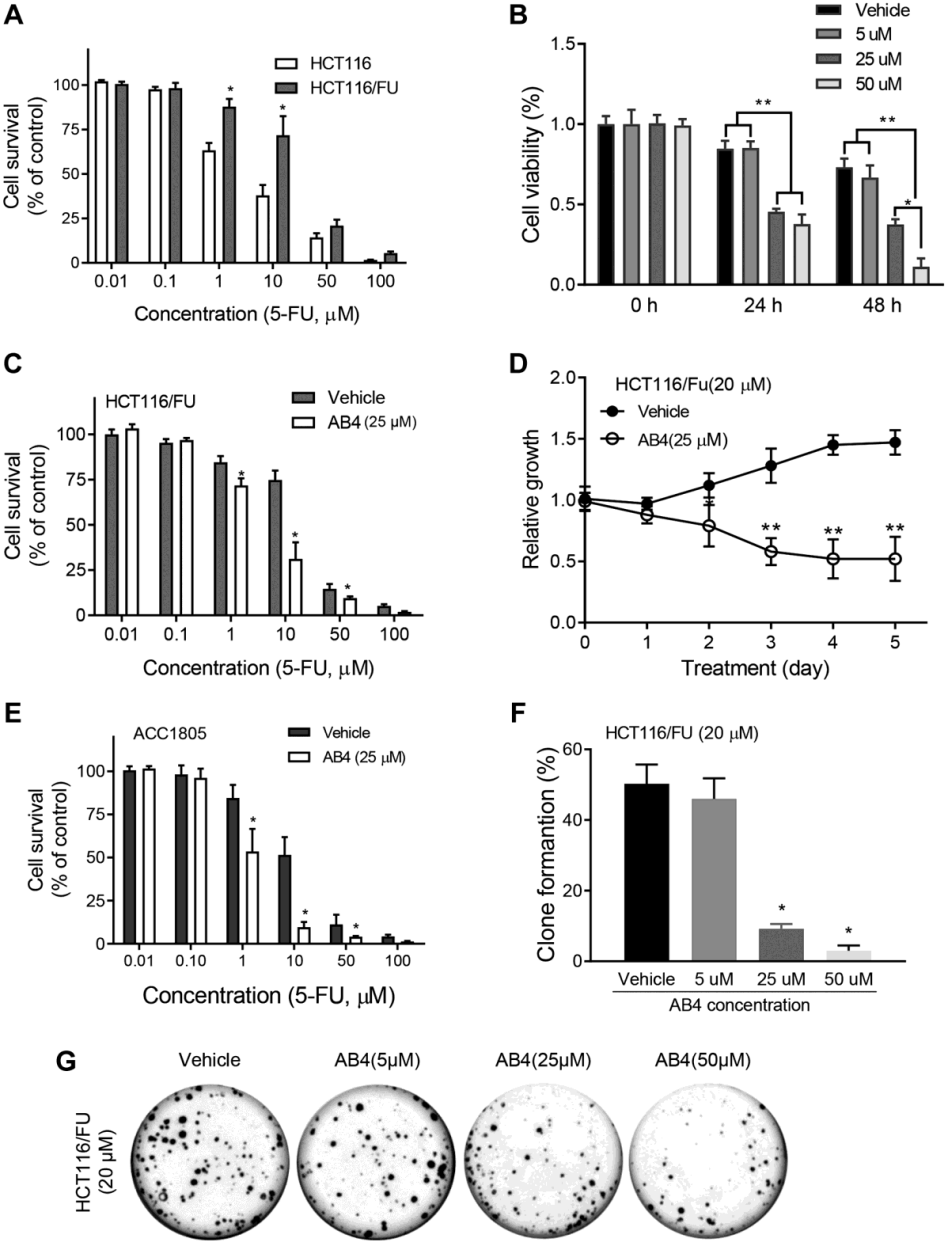
**AB4 sensitizes colorectal cancer cells to 5-FU treatment *in vitro*.** (**A**) HCT116 and HCT116/FU cells were treated with a gradient concentration of 5-FU (0–100 μM), which showed increased cell survival of HCT116/FU cells than parental cells. (**B**) HCT116/FU cells were treated with 5-FU (20 μM) and different concentration of AB4 (5–50 μM) for 24 or 48 hours. Cell viability was measured by MTT assays. (**C**, **E**) HCT116/FU and ACC1805 cells were treated with 25 μM AB4 and a gradient of 5-FU for 48 h. The cell proliferation was analyzed. (**D**) HCT116/FU cells were treated with AB4 (25 μM) and/ or 5-FU (20 μM) for 5 days. Cell proliferation was examined with MTT assay. (**F**, **G**) Colony formation assays were performed with HCT116/FU cells, which showed reduced number of colonies in AB4 treated group (≥25 μM) than vehicle or 5 μM AB4 treated groups. These results represent the average of three independent experiments. Data are shown as means ± SEM. ^*^P < 0.01.

FU resistant colorectal cancer cells, HCT116/FU cells were prepared with gradient elevated concentration of 5-FU for six months. HCT116/FU cells showed increased cell survival than the parental cells with MTT assays (Figure 1A). Then we evaluated the antiproliferative effects of combined treatment of 5-FU and a gradient of AB4 in HCT116/FU cells by MTT assays. The cell viability analysis revealed that combined treatment of AB4 (≥25 μM) notably restrained the proliferation of HCT116/FU cells (Figure 1B). Further studies demonstrated significantly decreased cell survival in 5-FU resistant HCT116 cells with combined 25 μM AB4 treatment than 5-FU only (Figure 1C). Notably, 25 μM AB4 significantly inhibited the proliferation of HCT116/FU cells in 5 days treatment, which indicated AB4 reversed 5-FU resistance *in vitro* (Figure 1D). Similar results were observed in AB4 treated ACC1805 cells, a primary colorectal cancer cell (Figure 1E). Colony formation assay was performed to measure the long-time survival inhibition effects of AB4 treatment. As expected, cell survival rate was reduced obviously in AB4 treatment groups in a dose-dependent manner (Figure 1F), which showed reduced number of cell colonies in AB4 treated group (≥25 μM) than other ones (Figure 1G). These results supported AB4 sensitizes chemotherapy resistant colorectal cancer to 5-FU *in vitro*.

### AB4 eliminates colorectal cancer stem cells

**Figure 2:**
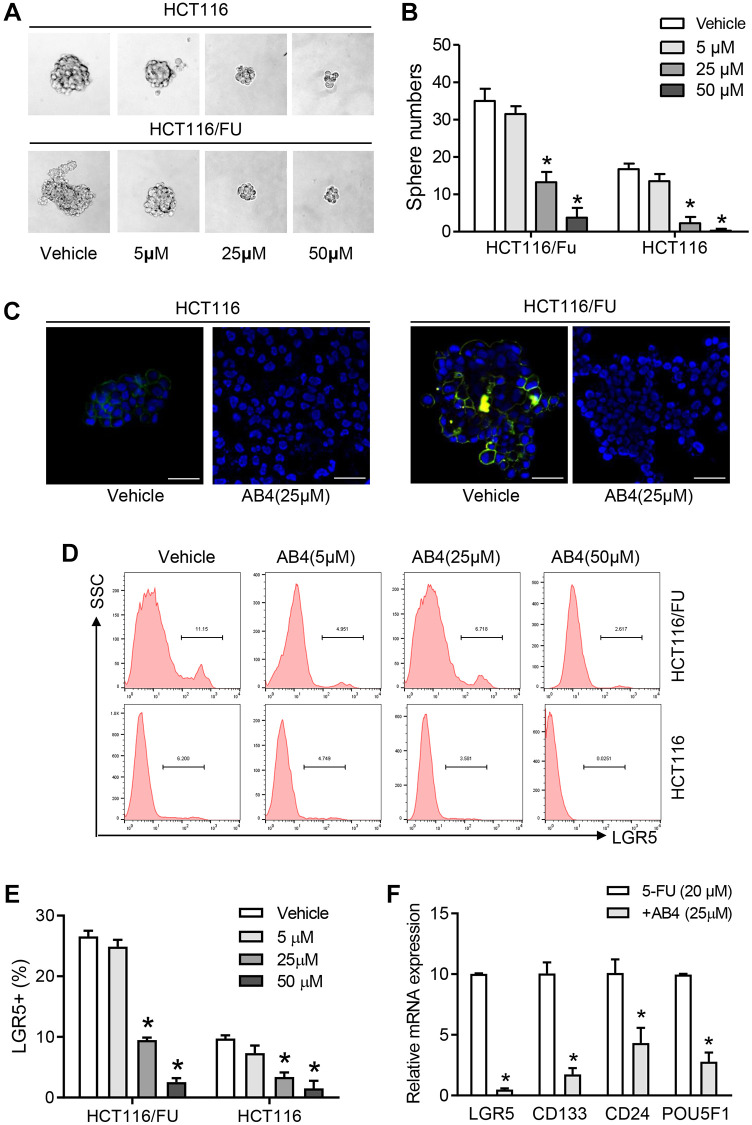
**AB4 eliminates colorectal cancer stem cells.** (**A**) Totally 1000 single colorectal cancer cells (HCT116 and HCT116/FU) were cultured in suspension culture system with gradient AB4 treatment (0, 5, 25, 50 μM). Representative images of tumor cell spheres were captured in the 10th day. (**B**) The numbers of tumor spheres at the tenth day were counted and compared between groups. (**C**) Confocal assays were performed for the expression of LGR5 (Green) in HCT116 and HCT116/FU cells, which was treated with AB4 for 24 days. Nuclei (blue). (**D**, **E**) The percentage of LGR5^+^ cells of HCT116/FU and HCT116 were analyzed with flowcytometry, which were treated with different concentration of AB4 (0, 5, 25, 50 μM) for 48 h. (**F**) Relative mRNA expression of cancer stem cell-related genes in AB4 (25 μM)/5-FU (20 μM) treated HCT116/FU cells and only 5-FU treated cells. Data represent three independent experiments, and shown as the means ± SEM. ^*^P < 0.01.

Further studies were performed for the stemness related traits of AB4 treated 5-FU resistant colorectal cancer cells. Our studies indicated that AB4 treatment suppressed tumor sphere formation in suspension culture of HCT116 and HCT116/FU cells, which showed decreased sphere volumes in AB4 treated cells than the control groups (Figure 2A). A significant decrease in the number of sphere-forming cells indicated a reduced self-renewal capacity of the AB4 treated cells (AB4 > 25 μM, Figure 2B). Immunofluorescent staining with HCT116/FU and HCT116 sphere cells indicated that AB4 treatment decreased the expression levels of LGR5 (Figure 2C), which was an important biomarker for colorectal cancer stem cells. Furthermore, after 48 h culture with gradient concentrations of AB4, the percentage of LGR5^+^ or CD133^+^ cells were significantly decreased with flowcytometry analysis (AB4 > 25 μM, Figure 2D, 2E). Further PCR analysis also indicated decreased mRNA levels of cancer stem cell biomarkers in 25 μM AB4 treated HCT116/FU sphere cells, including LGR5, CD133, CD24 and POU5F1 (Figure 2F). Taken together, these data suggested that AB4 was an effective agent in inhibiting self-renewal capacity of colorectal cancer cells.

### AB4 promotes apoptotic cell death of 5-FU resistant colorectal cancer cells

**Figure 3:**
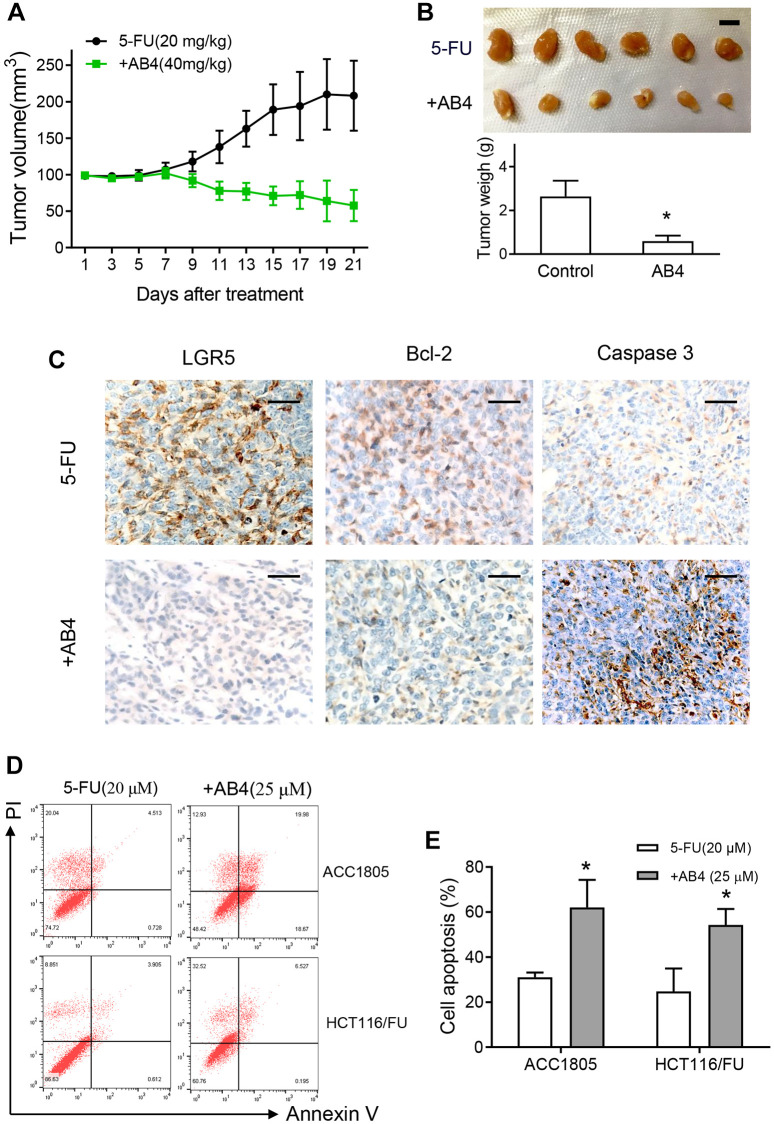
**AB4 promotes apoptotic cell death of 5-FU resistant colorectal cancer cells.** (**A**) Xenografts of HCT116/FU cells were implanted subcutaneously until the volume was about 100 mm^3^ in nude mice. The mice were treated with intravenous infusion of 5-FU (20 mg/kg) or combined with AB4 (40 mg/kg) every three days. Tumor volume was measured in the indicated day. The curves showed the mean volume ± SEM of tumor size. (**B**) Xenografts were harvested and imagined after 21 days treatment. The tumor volumes were compared between groups. Bar = 1 cm. (**C**) Representative IHC staining images of the expression of LGR5, bcl-2 and caspase 3 in the xenografts of different group. Bar, 50 μm. (**D**) HCT116/FU and ACC1805 cells were treated with 25 μM AB4 and 20 μM 5-FU for 48 h. Cells were collected and stained for cell apoptotic analysis by flow cytometry. (**E**) Statistical analysis of cell apoptosis in different groups as described in (**D**). The results represent three independent experiments. Data are shown as means ± SEM. ^*^P < 0.01.

We further investigated the therapeutic efficacy of AB4 on tumor growth of colorectal cancer *in vivo*. Dissociated HCT116/FU cells were subcutaneous implanted into athymic nude mice until the xenograft was about 100 mm^3^. Then the mice were treated with intravenous infusion with 5-FU and AB4 or vehicle every three days. Tumors in AB4-treated mice showed decreased tumor growth compared to only 5-FU treated ones (Figure 3A). Moreover, AB4-treated tumors showed significantly lower volume and weight than the control group (Figure 3B). IHC staining was performed with the xenografts, which showed AB4 treatment decreased LGR5 expression in xenografts (Figure 3C). Notably, decreased Bcl-2 and increased Caspase-3 expression was observed in the AB4 treated tumors (Figure 3C). Furthermore, flowcytometry analysis was performed to measure cell apoptosis with AB4 treatment. Compared to vehicle control group, combined AB4 treatment significantly increased early and late apoptosis percentages of HCT116/FU and ACC1805 cells, a primary colorectal cancer cell line (*p* < 0.01, respectively. Figure 3D, 3E). Collectively, these results indicated that AB4 promoted apoptotic cell death of 5-FU resistant colorectal cancer cells.

### AB4 activates caspase-9 pathway in 5-FU resistant colorectal cancer cells

**Figure 4:**
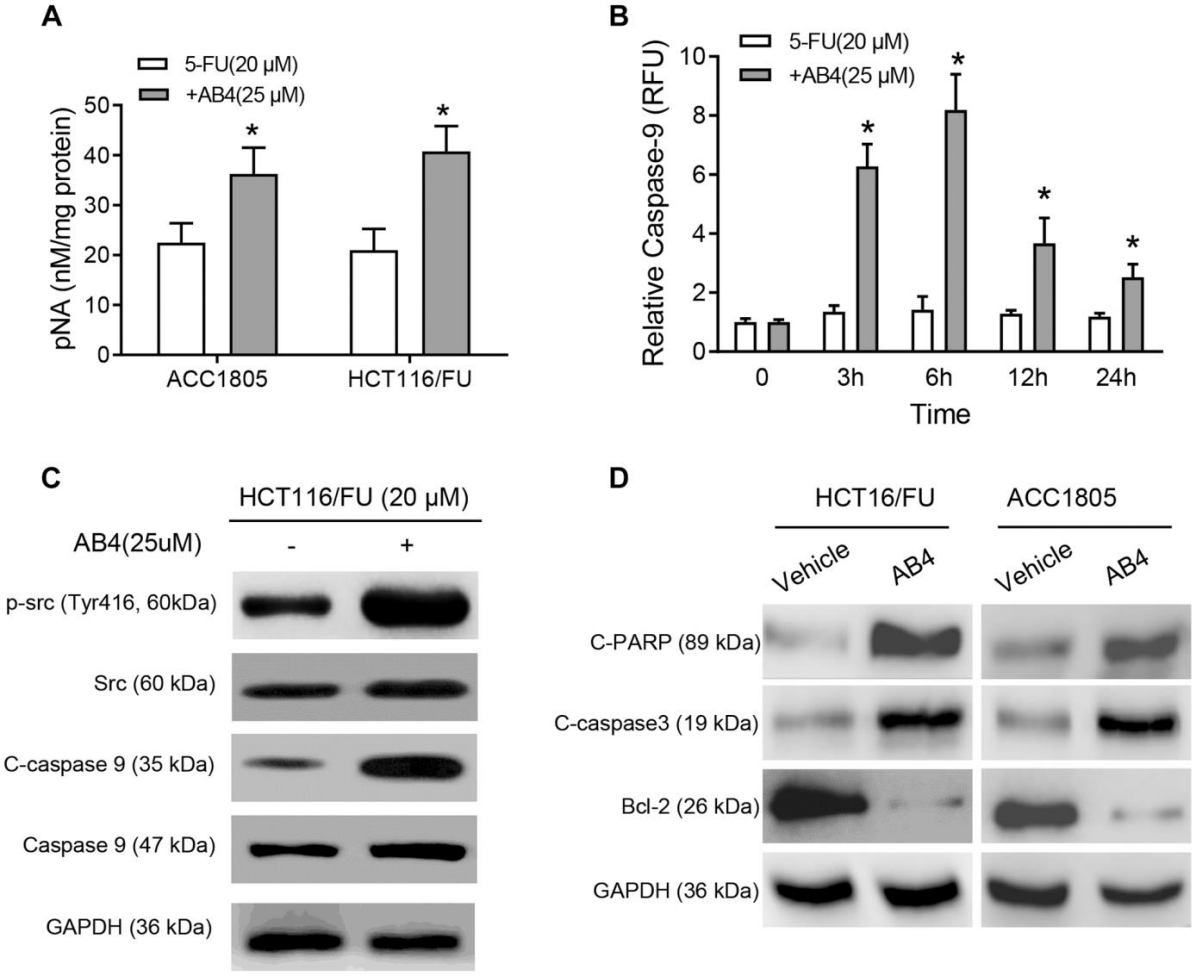
**AB4 activates Caspase 9 pathway in 5-FU resistant colorectal cancer cells.** (**A**) HCT116/FU and ACC1805 cells were treated with 25 μM AB4 and 20 μM 5-FU for 48 h. Control groups were treated with 5-FU only. Activity of caspase 3 was measured with pNA concentrations. (**B**) HCT116/FU cells were treated with 25 μM AB4 and 20 μM 5-FU. The activity of caspase-9 was measured and compared between groups in 0, 3, 6, 12, 24 hours. (**C**) HCT116/FU cells were treated with 20 μM 5-FU and/or 25 μM AB4. Western blot assays were performed for the Src-caspase-9 signaling pathway activation status. (**D**) Apoptosis related proteins (cleaved PARP, caspase 3, Bcl-2) were measured with Western blot assays. The results represent three independent experiments. Data are shown as means ± SEM. ^*^P < 0.01.

Based on the elevated cell apoptosis of AB4 treated tumors, we further analyzed the molecular mechanism of AB4 facilitated 5-FU-sensitivity of colorectal cancer cells. HCT116/FU and AAC1805 cells were treated with 5-FU and AB4 combined treatment. Correspondingly, increased caspase 3 activities were observed in combined AB4/5-FU treatment than only 5-FU (*p* < 0.01, respectively. Figure 4A). Furthermore, HCT116/FU cells also indicated increased caspase-9 activity with the combined 5-FU/AB4 treatment than only 5-FU especially after 3 hours treatment (*p* < 0.01, respectively. Figure 4B). Notably, Western blot assays were performed for the activation of Src-caspase-9 signaling pathway, which indicated that AB4 treatment increased Src phosphorylation and cleaved caspase-9 (Figure 4C). Furthermore, the pro-apoptotic effects of AB4 were also supported by the increased cleavage of PARP and caspase 3, as well as reduced Bcl-2 expression in AB4/5-FU treated HCT116/FU and ACC1805 cells (Figure 4D). These results indicated that AB4/5-FU combined treatment conferred more chemotherapy induced apoptosis in colorectal cancer cells *via* activation of caspase-9-caspase 3-dependent apoptotic pathway.

### Src promotes synergistic effects of AB4 with 5-FU treatment

**Figure 5:**
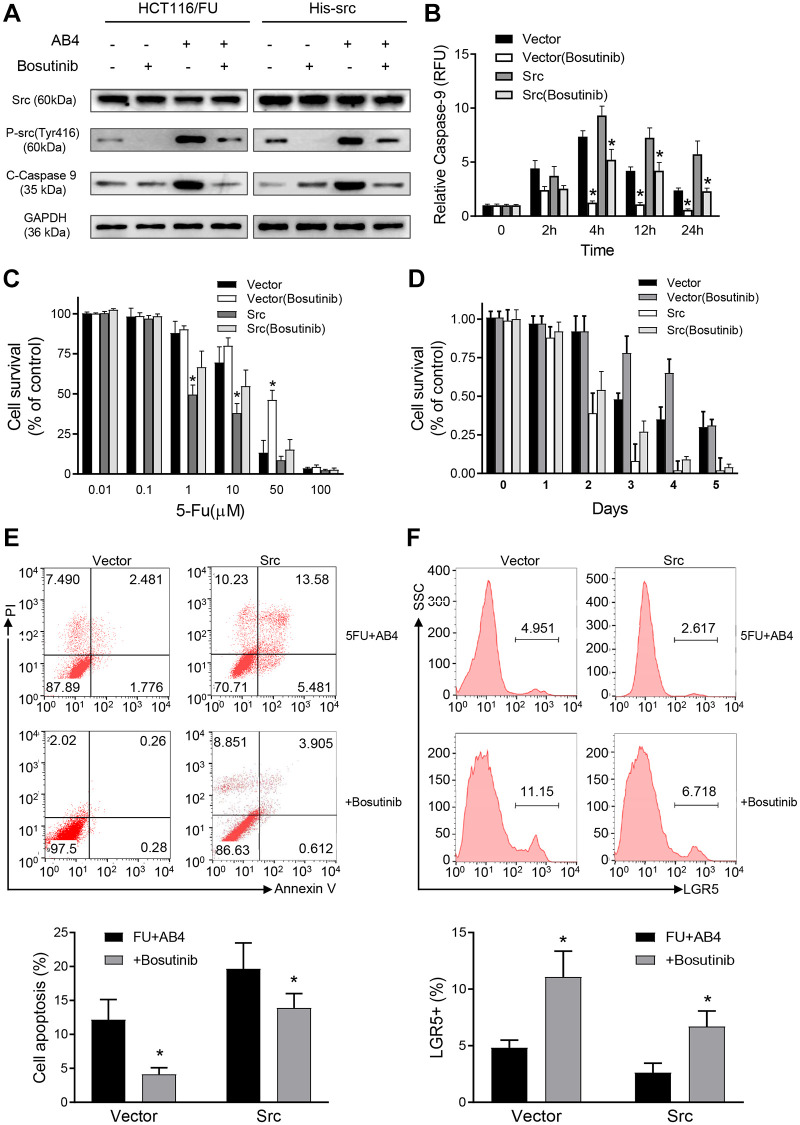
**Src promotes synergistic effects of AB4 with 5-FU treatment.** (**A**) HCT116/FU cells were transfected with His-Src or vector plasmids, followed by 25 μM AB4 and 20 μM 5-FU treatment. Bosutinib was used for Src signaling inhibition as indicated. Phosphorylated Src and cleaved caspase-9 protein were measured with Western blot assays. GAPDH was used as loading control. (**B**) Infected HCT116/FU cells were treated as indicated. Caspase-9 activity was evaluated in the indicated time. (**C**) MTT assays were performed with the infected HCT116/FU cells with gradient of 5-FU. HCT116/FU-vector cells were also treated with Bosutinib for Src signaling inhibition. (**D**) Infected HCT116/FU cells were treated with 25 μM AB4, 20 μM 5-FU and Bosutinib for 5 days. Cell viability was examined by MTT assays. (**E**) Infected HCT116/FU cells were treated with AB4,5-FU and Bosutinib. Cell apoptosis was analyzed by flow cytometry. (**F**) The percentage of LGR5^+^ of HCT116/FU-Src and control cells were analyzed by flowcytometry, which were also treated with 25 μM AB4 for 48 h. The results represent at least three independent experiments. Data represent as the means ± SEM. ^*^P < 0.01.

In order to assess the role of Src protein on the synergistic effects of AB4 and 5-FU treatment, HCT116/FU cells were transfected with His-Src or vector plasmids, followed by AB4/5-FU treatment and evaluation of the apoptotic pathway. Bosutinib was used for the inhibition of Src signaling pathway. Increased cleavage of caspase-9 was detected in the His-Src overexpressing cells with AB4 treatment, whereas dramatically decreased in Bosutinib treated HCT116/FU-vector or HCT116/FU-Src cells (Figure 5A). Our results indicated that Src phosphorylation contributed to AB4 induced caspase-9-related apoptotic pathway. Meanwhile, Src overexpression increased caspase-9 activity with AB4 treatment, and Bosutinib attenuated the caspase-9 activity in AB4 treated HCT116/FU cells (Figure 5B). MTT assays were performed to detect cell proliferation inhibition effects of 5-Fu in colorectal cancer cells. Combined treatment with Bosutinib increased cell survival rate, whereas decreased cell survival in Src-overexpressing cells than control cells (Figure 5C). Moreover, Src overexpression increased 5-FU/AB4 induced cytotoxicity compared to corresponding control cells, while Bosutinib reduced the cytotoxicity effects of the combined treatment (Figure 5D). Increased cell apoptosis was observed in Src-overexpressed cell than the control cells with combined AB4/5-FU treatment, and Bosutinib decreased cell apoptosis (Figure 5E). Flow cytometry analysis also showed lower percentage of LGR5^+^ cells in HCT116/FU-Src cells than control cells after AB4 treatment, whereas Bosutinib attenuated the cancer stem cell elimination effects of AB4/5-FU co-treatment (Figure 5F). Together, the above findings implicated that Src was involved in modulating apoptosis by AB4/5-FU induced caspase-9 activity. Moreover, Src-caspase-9 signaling was a promising target for colorectal cancer stem cell elimination.

## DISCUSSION

Our study indicated that AB4 displayed synergistic activity with 5-FU treatment in colorectal cancer cells. More importantly, AB4 treatment reversed 5-FU resistance and eliminated cancer stem cells of colorectal cancer. Mechanically, we found that AB4 enhanced colorectal cancer cell apoptosis through Src-dependent caspase-9 apoptotic pathway. Increased Src activation induced caspase-9 phosphorylation to sensitize 5-FU cytotoxicity. Our findings shed light to the mechanism study of 5-FU chemotherapy resistance, which provided a novel agent to ameliorate FU-based chemotherapeutic efficiency in colorectal cancer.

AB4 is one of the major active chemical ingredients in *P. chinensis* [11]. Previous studies supported its activity in anti-inflammatory and immune-modulatory properties [13]. AB4 treatment induces tumor cell apoptosis, autophagy and angiogenesis inhibition [12, 16]. In this study, we found a significantly synergistic effect of AB4 in FU-based chemotherapy of colorectal cancer *in vitro* and *in vivo*. Combined treatment of AB4/5-FU showed increased cell apoptosis. Furthermore, AB4 reversed 5-FU resistance of colorectal cancer cells, which is a promising way to overcome chemotherapy resistance of colorectal cancer. Further clinical trials should be performed for standard medication of AB4 for colorectal cancer patients.

Chemotherapy resistance is an important challenge for colorectal cancer, especially for the patient with metastatic colorectal cancer [17]. FU is an antimetabolite to suppress pyrimidine synthesis by thymidylate synthetase inhibition. Nucleoside metabolism obstacle induces cell death in tumor cells [3]. Endogenous caspase apoptotic pathway plays a crucial role in DNA damage in chemotherapy [18]. Chemotherapy resistant cells exhibit decreased cell apoptosis and DNA damage with FU treatment [19], as well as the signaling blockage of caspase apoptosis pathway [20]. Previous studies indicated that the anti-cancer effects of AB4 was correlated with the inhibition of PI3K/Akt/mTOR pathway in hepatocellular carcinoma [12]. Of note, our study indicated that 5-FU resistant cells showed decreased caspase-9 activity, whereas AB4 treatment rescued its activity in a dose-dependent manner. Moreover, AB4 showed protective effects against cisplatin-induced nephrotoxicity without reducing anti-tumor activity [15]. Therefore, combined treatment with AB4 will be a promising way to enhance therapeutic efficiency and to reverse chemotherapy resistance. Further clinical trials are needed for the synergistic activity of AB4 with conventional chemotherapy.

As the main initiating caspase member of endogenous apoptosis, phosphorylated caspase-9 shows enhanced activity. Increased caspase-9 phosphorylation is observed in Src kinase co-expressing cells [5]. Src kinase contributes to the metastatic phenotype of colon cancer cells [21], which is involved in the carcinogenesis and cancer progression [22, 23]. However, previous studies also proved Src kinase directly phosphorylate caspase-7 and 9 [9]. Notably, Src-dependent caspase-9 apoptotic pathway plays a key role in chemotherapy response. Mechanically, we proved that AB4 treatment enhanced Src kinase activity, subsequently Src kinase increased caspase-9 activity by phosphorylation to promote cell apoptosis. However, further studies are still needed for the genomic influence of AB4 treatment in colorectal cancer cells, and that not limitation to cell apoptosis.

Accumulating evidence supported that cancer stem cells contributed to chemotherapy resistance [24, 25]. The biological trait of continuous self-renewal and differentiation grants them survival advantage in toxic internal environment [26]. Enriched cancer stem cells are observed in chemotherapy resistant cancer bulks [25, 27]. Cell apoptotic pathway blockage is identified to be critical for cancer stem cell maintenance [28, 29]. Previous studies indicated several dietary compounds potentially eliminate cancer stem cells, such as curcumin and epigallocatechin-gallate [30, 31]. In this study, we found AB4 eradicated colorectal cancer stem cells to reverse chemotherapy resistance. Caspase 9 cell apoptotic pathway played a crucial role in the cancer stem cell elimination effects. Thus, targeting cell apoptotic pathways may provide an effective strategy to eliminate cancer stem cells and thereby overcome tumor resistance to reduce relapse.

Taken together, Chinese herbology provides a promising new source for oncotherapy. Among them, AB4 exhibits a broad applying prospect in colorectal cancer management. Our study indicated the synergistic effects of AB4 in 5-FU chemotherapy of colorectal cancer. More importantly, AB4 sensitizes human colorectal cancer to fluorouracil-based chemotherapy through Src-dependent caspase-9 apoptotic pathway. Our study provided a way to ameliorate FU-based chemotherapeutic efficiency for colorectal cancer treatment.

## MATERIALS AND METHODS

### Cell culture and transfection

HCT116 cells were obtained from ATCC and cultured in DMEM, containing 10% fetal bovine serum. ACC1805 was a primary cell line from colorectal cancer malignant ascites in a patient whom developed resistance to 5-FU. 5-FU and AB4 were obtained from Sigma-Aldrich (St Louis, MO). The 5-FU-resistant HCT116/FU cells were developed from the parental HCT116 cells which were subjected to persistent gradient exposure to 5-FU for about 6 months, through increasing 5-FU concentration from 0.05 μg/ml to 1 μg/ml. Bosutinib (SC1004) was obtained from Beyotime Biotechnology (Shanghai, China). Full-length human Src was PCR-amplified with primers (forward primer: GCTTGGTACCGAGCTCGGATCCACCATGGGTAGCAACAAGAGCAAG, Revers primer: TGCTGGATATCTGCAGAATTCTCAATGGTGATGGTGATGATGGAGGTTCTCCCCGGGCTGGT) from the pDEST40-2XFL-wtSrc plasmid (a gift from Mark Moasser, Addgene plasmid #140295). His-Tag was added to C-terminal of human Src and subcloned into plasmid pcDNA3.1(+) (Invitrogen) between the site of BamH I and EcoR I according to the Gibson Assembly Cloning Kit protocol (NEB, E2611L). Plasmid transfection of His-Src and vector plasmids was performed with Lipo6000™ Transfection Reagent (C0526. Beyotime, China) according to the standard protocols.

### Western blot analysis

Western blot was performed as previous report [32]. Primary antibodies against total Src (#2108), Phospho-Src (Tyr416, #6943), cleaved PARP (#9548), cleaved caspase 3 (#9661), caspase-9 (#9502) and cleaved caspase-9 (#9505) were obtained from Cell Signaling Technology (Boston, MA). Antibodies against bcl2 (ab32124), GAPDH (ab181602) and His (ab18184) were obtained from Abcam (Cambridge, UK).

### Caspase-9 activity analysis

Caspase-9 Activity Assay Kit (Beyotime, Shanghai, China) was used to measure caspase-9 activity. After collection of whole-cell lysates in each group, 200 μg of protein was utilized to assess the caspase-9 activity in accordance with standard protocols.

### MTT assays

MTT Cell Proliferation and Cytotoxicity Assay Kit (Beyotime) was used to estimate cytotoxicity. Cells were placed in 96-well plates (2000 cells per well) and exposed to indicated treatment. Cells were incubated with MTS for 1 h at 37°C in an incubator containing 5% CO_2_, followed by absorbance at a wavelength of 492 nm. The IC50 values were measured through non-linear regression analysis by GraphPad Prism software (GraphPad Software Inc., San Diego, CA).

### Colony formation assays

Single cells were seeded in six-well plates (1000 cells per well) and cultured for 14 days. Cells were cultured with fresh medium and corresponding treatment every other day. Crystal violet was used to stain the cell colonies. Colonies formed by more than 50 cells were counted. The numbers of colony were shown as mean ± SEM. from at least three independent experiments.

### Tumor sphere formation assays

Totally 1000 cancer cells were seeded in a 24-well ultralow attachment plate with serum-free DMEM/F-12 medium (including 20 ng/ml EGF, 20 ng/ml bFGF and 2% B27). Cells were cultured with the indicated treatment for ten days, then counted manually by inverted phase contrast microscopy and compared between groups.

### Immunofluorescence analysis

Tumor sphere cells were cultured on glass coverslips in 6-well plates, and AB4 was added to the culture media for 48 hours and then fixed in a solution of 4% paraformaldehyde in PBS for 30 minutes. Then permeabilized with 0.1% Triton X-100 phosphate-buffered saline (PBS) for 10 minutes, and blocked in 5% goat serum in PBS for 1 hour. LGR5 antibody was incubated overnight at 4°C. Then washed and incubated with the secondary antibody for 1.5 hour at 37°C. All images were collected using a confocal Zeiss LSM780 microscope (Zeiss, Germany).

### Annexin V-fluorescein isothiocyanate (FITC) apoptosis analysis

Cell apoptosis were measured with Annexin V-FITC Apoptosis Detection Kit (Beyotime) according to the manufacturer’s instructions. Cells were harvested and washed with PBS. Then incubated with annexin V-FITC and propidium iodide for 15 min at room temperature. Prepared cells were analyzed with FACS Calibur flow cytometer (BD Biosciences, San Jose, CA).

### Flow cytometric analysis of LGR5

Prepared cells were collected and incubated with LGR5 primary antibody (ab75732, Abcam) in 4°C for 30 minutes. Then washed and incubated goat anti rabbit Alexa Fluor 647 antibody (Sparks, MD) at 4°C for 30 min. Cells were washed and measured with FACS Calibur flow cytometer (BD Biosciences).

### Tumor xenograft models

Prepared cells were subcutaneously injected in upper flank region of athymic nude mouse. Tumor volumes were measured with caliper and calculated as (length × width^2^)/2 which. Therapeutic experiments were started when the tumors were approximately 100 mm^3^. Mouse were treated with intravenous infusion of 5-FU (20 mg/kg) or combined with AB4 (40 mg/kg) every three days. Tumor volume was measured in the indicated day. The curves showed the mean volume ± SEM of tumor size at different time points. The animal experiments were approved by the Institutional Animal Care and Use Committee of the 901 Hospital of Joint Logistics Support Force. The animal experiments were compliant with the animal care guideline of our hospital.

### Immunohistochemistry assays

Immunohistochemistry assays were performed as previous report [32]. Primary antibodies against LGR5, bcl-2, and caspase-9 were obtained from Abcam. Deparaffinized xenograft sections were prepared and stained with with Ventana Discovery XT automated staining system (Ventana Medical Systems, Inc., Tucson, AZ).

### Statistical analysis

All experiments were independently performed at least 3 times. All quantitative data were presented as mean ± SEM and analyzed with Student’s *t* test. Statistical significance among all groups (*n* ≥ 3) was determined with one-way analysis of variance. The results were analyzed with GraphPad Prism 5 (GraphPad Instat Software, La Jolla, CA). *P* < 0.05 was considered as statistically significant.
